# Community-based participatory research in a “Shitamachi” neighborhood in Tokyo: building social capital and relational spaces for community health

**DOI:** 10.1186/s12889-026-26475-5

**Published:** 2026-02-11

**Authors:** Daisuke Son, Toshichika Mitsuyama, Yuzuki Matsushita

**Affiliations:** 1 Department of Community-based Family Medicine, Faculty of Medicine, Tottori University, Yonago, Tottori, 683-8503 Japan; 2 Department of Medical Education Studies, International Research Center for Medical Education, Graduate School of Medicine, The University of Tokyo, Bunkyo-ku, Tokyo 113-0033 Japan; 3 Medical Corporation Kagayaki General Home Care Clinic, Hashima-gun, Gifu, 501-6014 Japan; 4 Fukushozan Houzen-in Temple, Hiratsuka, Kanagawa 254-0052 Japan

**Keywords:** Community-Based participatory research, Urban health, Social capital, Relational well-being, Mobile yatai

## Abstract

**Background:**

Community-Based Participatory Research (CBPR) offers a relational framework for bridging scientific inquiry and everyday life. Yet its adaptation to Japan’s urban contexts—where dense traditions coexist with social fragmentation—remains underexplored.

**Methods:**

Since 2015, the YaNeSen CBPR has linked residents, physicians, and researchers in Tokyo’s historic Shitamachi district of Yanaka–Nezu–Sendagi to co-create health-promoting spaces grounded in local culture. Using ethnographic fieldwork, community asset mapping, and collaborative action, the team developed the “Mobile Yatai de Health Café”—a movable wooden stall that was intermittently deployed between 2016 and 2020 to serve coffee and invite spontaneous street-level dialogue between residents and care professionals. Follow-up interviews with 12 participants in 2021 were thematically analyzed to assess long-term transformations.

**Results:**

Early engagement revealed temples, public baths, and alleys as “third places” sustaining social capital. The Mobile Yatai extended this ecology by generating salutogenic encounters that blurred boundaries between health, art, and everyday sociability. Interview narratives described the project as a “space of relational invitation” (kakawari-shiro) characterized by openness, serious play, and an ethics of non-obligation. Participants reported a broadened sense of health as relational, expressive, and experiential rather than biomedical.

**Conclusions:**

The YaNeSen CBPR demonstrates how culturally embedded, art-based collaboration can nurture “relational commons” that sustain well-being in aging urban communities. By valuing presence, ambiguity, and care over prescriptive intervention, this study reframes health as convivial coexistence through culturally embedded participatory art.

## Introduction

Community-Based Participatory Research (CBPR) has emerged globally as a transformative approach to bridging the gap between academic research and community life. Rather than positioning communities as mere subjects of study, CBPR emphasizes equitable partnership and co-learning among researchers, practitioners, and local residents [1, 2]. Over the past three decades, it has become a key methodology for addressing health disparities and fostering social inclusion, particularly in marginalized populations. By aligning research agendas with local priorities, CBPR enhances both the relevance and the sustainability of community health interventions [2, 3]. 

While CBPR has gained significant traction in North America, Europe, and parts of Asia, its adaptation to Japan’s socio-cultural context remains underexplored. Japanese society is characterized by strong community traditions and dense social networks, yet contemporary urbanization and demographic aging have eroded many of these relational infrastructure [4, 5]. In rural areas, CBPR has often been embedded in administrative programs for community medicine or public health promotion [6–8]. However, in metropolitan settings—where traditional neighborhood ties coexist with transient populations—CBPR’s relational and cultural dimensions have received little empirical attention. The question arises: How can participatory research foster health and belonging in complex, culturally layered urban environments?

The Yanaka–Nezu–Sendagi (YaNeSen) district in Tokyo offers a distinctive lens through which to examine this question. Situated on the fringe of Ueno Park, the area has long been known as “Shitamachi”—Tokyo’s traditional downtown—where craftspeople, small merchants, and temple communities coexist within narrow alleys and wooden row houses. Despite rapid gentrification since the 1990s, YaNeSen maintains a deep sense of place and identity, mediated by cultural continuity and collective memory. Grassroots initiatives such as the Geikoten art festival and the Yanaka–Nezu–Sendagi magazine have served as cultural archives, sustaining everyday civic engagement through storytelling and art. Scholars have described Shitamachi neighborhoods as “moral communities” [9] where local interactions embody trust, reciprocity, and shared ethics. Yet few studies have examined how such cultural ecologies can be leveraged to reimagine health and well-being.

In this context, the YaNeSen CBPR project was initiated in 2015 as a long-term collaboration between community members, physicians, and researchers in public health and the humanities. The project sought not only to identify local health needs but also to co-create spaces of dialogue and expression that could nurture relational well-being. The initiative unfolded through multiple cycles of co-planning, collective action, and reflexive dialogue—an approach influenced by Freirean participatory pedagogy [10] and Japanese notions of *ba* (relational field) [11]. 

This paper presents the YaNeSen CBPR as an evolving three-phase process. The early phase (2015–2016) involved ethnographic engagement to map community assets and build trust through everyday encounters. The action phase (2016–2020) centered on the Mobile Yatai de Health Café—a mobile, handcrafted stall that brought healthcare professionals and residents together in the streets for spontaneous conversations and creative collaboration. The outcome phase (2021) evaluated the project’s longer-term impacts through follow-up interviews with residents and collaborators, illuminating how these participatory practices reshaped local understandings of health and well-being. By examining these interconnected phases, this study explores how CBPR can generate a relational commons—shared affective and cultural spaces where art, mobility, and everyday care converge to expand the meaning of health beyond biomedical boundaries.

## Methods

### Study design and setting

The project followed a CBPR framework grounded in iterative cycles of co-planning, action, and reflection [1]. The YaNeSen district—spanning Bunkyo and Taito wards—was selected for its distinctive “Shitamachi” cultural ecology, characterized by narrow alleys, long-established neighborhood associations, and a mix of traditional and creative residents. The project aimed to co-create community spaces that integrate local culture and health promotion.

### Participants and partnership formation

The core partnership consisted of ten members, including physicians, public health professionals, university researchers, and local residents active in NPOs or art collectives (aged 30–60 s; gender-balanced). From 2015 to 2019, trust was gradually built through informal meetings in cafés, temples, and shared events. Early activities included community asset mapping and hosting neighborhood dialogues to identify shared concerns around isolation and well-being.

## Data collection

### Early phase (2015–2016)

Ethnographic fieldwork combined semi-structured interviews, participant observation, and fieldnotes. Interviews (*n* = 18) with community leaders, shop owners, artists, and welfare workers followed a flexible guide addressing local collaboration, everyday health, and perceived community change. Observations took place during festivals, neighborhood cafés, and art projects, providing contextual understanding of relational dynamics.

### Action phase: the “Mobile Yatai” project (2016–2020)

During the second year of the project, the team was invited by local residents to participate in *Geikoten*, a citizen-organized art festival that has been held annually since 1993. In response, the group initiated the “Mobile Yatai de Health Café”, an experiment in creating mobile and relational spaces for dialogue on health and everyday life.

The project unfolded in two stages:

- Yatai Construction Workshop – In collaboration with local architects, craftspeople, and healthcare professionals, the team built a wooden mobile stall (yatai) in the streets of Yanaka. Passersby—residents, children, and even international tourists—stopped to observe and converse. The act of co-building generated spontaneous intergenerational participation and curiosity.
- Health Café on Wheels – Once completed, the yatai was pulled through the alleys of Yanaka, Nezu, and Sendagi during the two-week festival in 2016. Family physicians, community nurses, and medical students offered coffee and casual conversation to anyone they encountered. Many people engaged in these encounters, with topics ranging from local memories to illness narratives and neighborhood stories.

Following the 2016 Geikoten, the mobile yatai activities continued intermittently in the YaNeSen area through 2020. The team periodically brought the yatai back into the streets for community events, health-related gatherings, and spontaneous neighborhood interactions. These ongoing sessions reinforced the project’s relational presence in everyday urban life, allowing residents and professionals to reconnect in new contexts and sustain dialogue about health, care, and place.

### Outcome evaluation (2021 follow-up)

Between March and April 2021, twelve participants—including both residents and professionals—were individually interviewed online via videoconferencing platforms (45–90 min each). Participant characteristics, including age range, gender, role in the project, duration of involvement, and type of involvement, are summarized in Table 1. Participants were selected using purposive sampling to capture a range of roles and levels of engagement within the project. As with many CBPR studies, participants with sustained involvement and positive relationships with the project may have been more likely to participate. Interviews explored personal experiences, meanings of participation, and perceived transformations in the community. Beyond infection control measures, the COVID-19 pandemic constrained the frequency, scale, and spontaneity of in-person project activities and shaped the timing and format of the follow-up interviews. Consequently, all follow-up interviews were conducted online.

**Table 1:** Characteristics of interview participants (*n* = 12)

Participant ID	Age range	Gender	Role in project / occupation	Duration of involvement	Type of involvement
P1	50s	male	project staff / company employee	> 3 years	core organizer / regular participant
P2	30s	female	project staff / company employee	> 3 years	core organizer / regular participant
P3	30s	female	project staff / welfare professional	> 3 years	core organizer / regular participant
P4	30s	male	project collaborator / nurse	2–3 years	project collaborator / regular participant
P5	40s	female	project collaborator / physiotherapist	2–3 years	project collaborator / regular participant
P6	60s	female	project collaborator / writer	2–3 years	project collaborator / regular participant
P7	30s	male	project collaborator / architect	> 3 years	project collaborator / occasional participant
P8	30s	male	project collaborator / company employee	> 3 years	project collaborator / occasional participant
P9	60s	female	local resident / community-based practitioner	> 3 years	project advisor / occasional participant
P10	30s	female	local resident / café owner	2–3 years	venue provider
P11	70s	male	local resident / café owner	2–3 years	venue provider
P12	30s	female	local resident / community-based practitioner	> 3 years	project advisor / occasional participant

“Role in project” refers to participants’ positional relationship to the CBPR initiative, while “Type of involvement” reflects the nature and frequency of their engagement over time

Participants interviewed in the 2021 follow-up were distinct from those interviewed in the early phase (2015–2016), with continuity across phases maintained at the level of project activities and community engagement rather than individual longitudinal follow-up.

Transcripts were analyzed thematically following Braun and Clarke’s six-phase framework [12]. The first and second authors independently conducted the initial coding, met regularly to compare interpretations, and refined overarching themes through iterative discussion. The third author then reviewed the coded data and thematic structure for triangulation, ensuring analytical consistency and interpretive rigor. Interviews were conducted until thematic saturation was reached, as no substantively new themes emerged in later interviews. Emerging interpretations were iteratively discussed within the research team and informally shared with participants during ongoing project activities as a form of member checking. NVivo 12 software facilitated data management. Reflexivity was maintained through researcher journals documenting positionality and evolving relationships.

### Reflexivity statement

All authors were actively involved in the design and implementation of the *YaNeSen* CBPR project and therefore occupied dual roles as researchers and participants. The first author is a family physician and medical educator with a longstanding interest in health humanities and community-based practice, which shaped an a priori sensitivity to relational, cultural, and experiential dimensions of health. The second author is also a family physician and medical education researcher, bringing a pedagogical and reflective lens to questions of professional identity, dialogue, and learning in community settings. The third author is a clinical psychologist and Buddhist priest, whose clinical and spiritual background informed attention to meaning-making, care, and ethical presence in everyday encounters.

These diverse positionalities placed the authors in partial insider roles within the project while also situating them as outsiders in relation to local residents. We acknowledge that these positions may have influenced data generation and interpretation, including a tendency to foreground relational and affirmative aspects of participation. To address this, reflexive journals were maintained throughout the study, and analytic decisions were regularly discussed among authors with differing professional backgrounds and degrees of involvement. Interview questions explicitly invited ambivalence, uncertainty, and critical reflections, and interpretations were repeatedly revisited in light of discrepant accounts. This reflexive stance aligns with CBPR principles emphasizing relational accountability and situated knowledge.

### Ethical considerations

This study was conducted in accordance with the ethical standards laid down in the Declaration of Helsinki. Ethical approval for this research was obtained from the Research Ethics Committee of the Faculty of Medicine, The University of Tokyo (Approval No. 10994). All participants received written and verbal information about the study aims and procedures and provided written informed consent prior to participation.

## Results

### 1. Early phase (2015–2016): emergent forms of social capital.

Initial fieldwork suggested multiple layers of social capital rooted in everyday spaces. Temples, public baths (sento), and renovated kominka (old houses) appeared to function as “third places” facilitating intergenerational contact. Local festivals, art workshops, and shared alley gardens were described by participants and observed in fieldwork as functioning as archives of community memory—sites where the history and identity of the neighborhood were continually reproduced.

Participants emphasized that these spaces contributed to psychological comfort and social inclusion. Challenges included the separation between long-term and newer residents, and limited engagement from younger families.

### 2. The Mobile Yatai as relational Intervention (2016–2020).

**Fig. 1:**
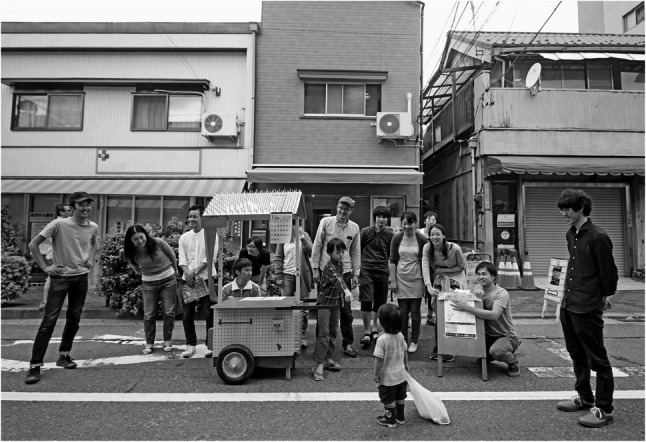
Mobile Yatai de Health Café at a neighborhood street festival in the YaNeSen district

**Fig. 2:**
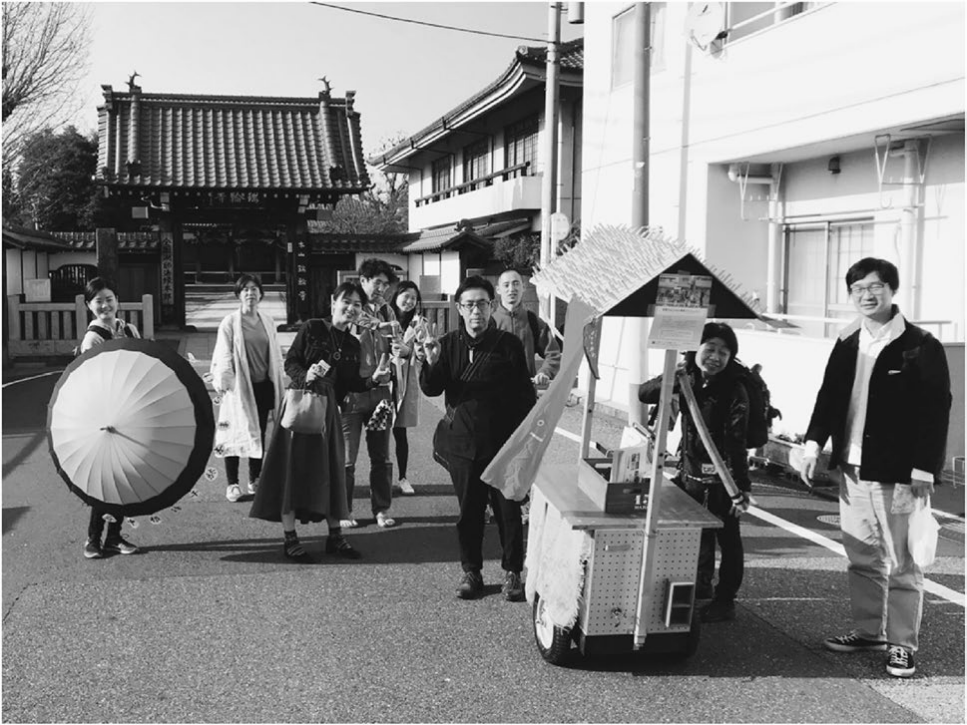
Mobile Yatai in front of a Buddhist temple

**Fig. 3:**
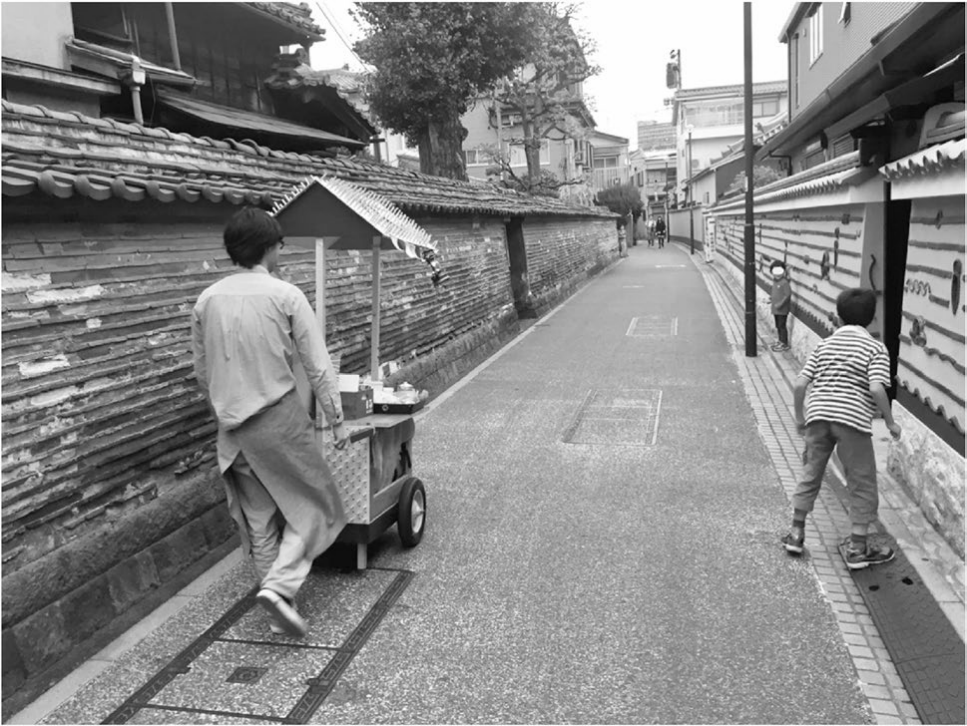
Mobile Yatai moving through a narrow residential alley

**Fig. 4:**
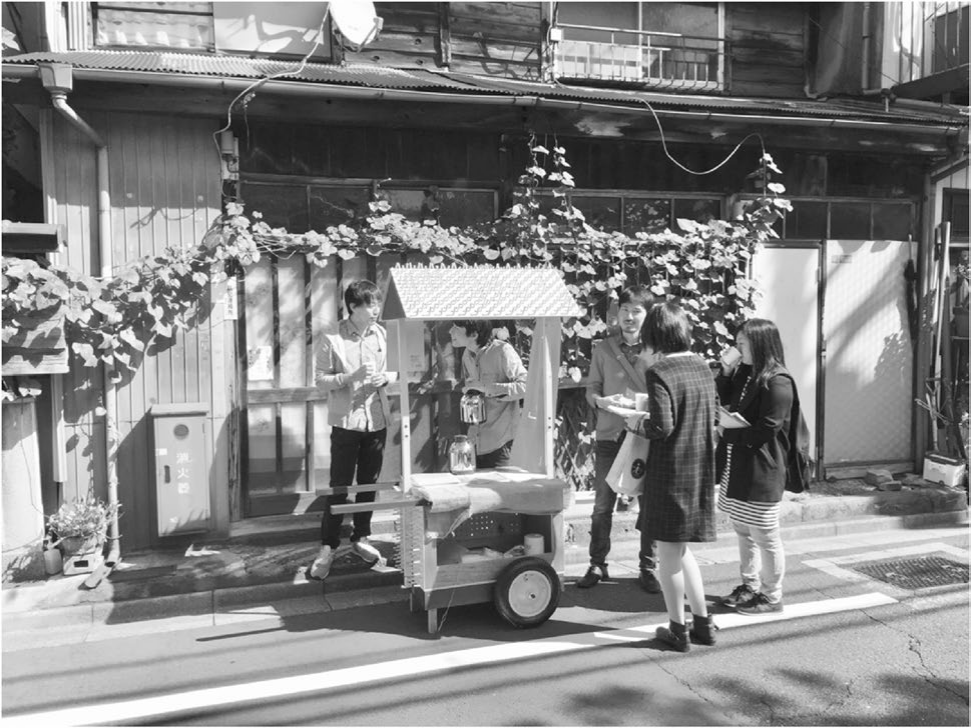
Conversations around the Mobile Yatai in front of a traditional house

The *M*obile Yatai project exemplified how CBPR could move from observation to action by transforming ordinary streets into arenas of salutogenic dialogue. Conversations were seldom centered on illness; instead, they often began with curiosity about the yatai itself—its design, the aroma of coffee, or shared nostalgia for disappearing public baths and neighborhood alleys (Figs. 1, 2, 3 and 4).

Fieldnotes captured scenes such as:

A woman in her 70s stopped and said, ‘Is this part of the art festival? Oh, doctors and monks together—that’s unusual!’ Soon several others gathered, chatting about local temples and family health.

Children joined in hammering wood panels, while their parents smiled and shared stories of the neighborhood.

Participants themselves articulated this boundary-blurring character of the activity. One participant reflected, “We don’t start by saying, ‘let’s talk about health.’ It’s the presence of things—food, objects, a place—that allows conversation to happen naturally.” Another noted that contemporary understandings of health have shifted away from life prolongation toward ways of being-with others in everyday life. The playful quality of the yatai was also repeatedly emphasized by participants. As one interviewee explained, “People who just come for fun are fine—actually, that makes it less awkward, and more people come.” Such remarks suggest that playfulness was not incidental but functioned as a condition that enabled participation without the pressure of health-related goals.

Taken together, these observations and participant reflections suggest that interactions around the yatai blurred boundaries between healthcare, art, and community life. Rather than positioning dialogue as an explicit health intervention, participants described how everyday, playful exchanges created conditions through which health-related meanings could emerge. The yatai’s mobility allowed engagement with residents who might otherwise avoid medical spaces, which participants described as fostering a sense of inclusivity and trust. Through the creative, sensory, and playful nature of the activity, the project illustrated what Antonovsky termed a salutogenic approach—focusing on factors that create health rather than prevent disease [13]. 

The Mobile Yatai during a neighborhood street festival held on a pedestrianized street in the *YaNeSen* district. Healthcare professionals, project staff from diverse backgrounds, local residents, and children gathered spontaneously around the stall. The scene captures the playful and convivial nature of the activity, as well as the blurring of boundaries between healthcare, community life, and cultural practice within an everyday urban setting.

The Mobile Yatai set up in front of a local Buddhist temple, where residents, project members, and passersby gathered informally. The scene illustrates how boundaries between healthcare, art, religious space, and everyday community life became blurred through convivial interaction in a non-clinical setting.

The Mobile Yatai moving through a narrow residential alley in the *YaNeSen* district. The mobility of the stall transformed the street into a site of spontaneous encounter, where children and residents engaged playfully with the project without prior invitation or fixed purpose.

Residents and project members conversing around the Mobile Yatai in front of a traditional house (kominka). Such everyday settings enabled multiple layers of social connection to emerge, including intergenerational dialogue and informal exchanges rooted in lived neighborhood spaces.

### 3. Outcome phase (2021): relational and experiential transformations.

#### CBPR as a Space of Relational Invitations (Kakawari-shiro).

This subsection presents themes grounded in participants’ own descriptions and expressions during interviews.

Participants described the CBPR project as an open, fluid field of relationships rather than a formally structured program. Entry points were casual—through encounters at temples, cafés, or public baths—lowering barriers to participation:

I didn’t really know who was doing what or why, but I was just there—that’s how it felt.

I was invited at a public bath—someone just said, ‘Want to help build a stall?’ and that’s how it started.

These accounts suggest experiences of belonging that were not explicitly tied to obligation, fixed roles, or predefined purposes. Rather than being organized around commitment or responsibility, participation unfolded through invitation, ambiguity, and improvisation, allowing the project to function as a relational platform for spontaneous collaboration.

### Blurring of expertise and everyday identity

Many participants valued how the CBPR space allowed professionals and residents to interact as equals:

Even without knowing each other’s background or profession, we looked out for one another and shared mutual respect.

It was actually good that we couldn’t tell who was the expert.

The ambiguity of roles dissolved traditional hierarchies, allowing personal identities to emerge beyond occupational status.

### “Serious play” and the ethics of non-obligation.

Respondents highlighted an atmosphere of serious play—deep engagement without pressure or institutional constraint:

People could come and go freely. It wasn’t overwhelming, but it still felt like a special place.

Everyone was seriously playing—but without attachment. That was the biggest appeal.

This “ethics of non-obligation” encouraged sustained participation through joy and curiosity rather than duty.

### Ambiguity and openness as conditions for trust

Finally, several respondents linked the project’s success to the Shitamachi culture of tolerance and improvisation:

Someone once said, ‘Do you really need a purpose?’ That gave me courage.

Because this town embraces ambiguity, those kinds of activities could be accepted.

Such cultural openness sustained trust and enabled experimentation without rigid evaluation criteria.

While participants did not use abstract terms such as “relational commons,” their accounts consistently described experiences of relational invitation, openness, invitation, and shared presence, which the authors synthesize in the Discussion using this conceptual framework.

## Discussion

In this study, health is understood not as an individual state to be measured or optimized, but as a relational and processual phenomenon that emerges through convivial co-existence, everyday interactions, and shared spaces within a community.

This study suggests that CBPR, when embedded in the everyday life of an urban neighborhood, can be interpreted as evolving into what might be called a relational commons—an analytic concept developed by the authors to refer to a shared social field where health, meaning, and belonging are co-created through interaction and imagination. In contrast to instrumental models of community health that emphasize measurable outcomes, the YaNeSen project foregrounds relational quality as an outcome in itself [14, 15]. The project’s longevity and continuity suggest that health promotion in complex urban contexts may require not only service provision but also the cultivation of spaces of invitation—what participants called kakawari-shiro—where people can encounter one another without fixed roles or expectations.

Borrowing in part from Gergen’s notion of “relational being,” [16] which emphasizes the co-constitutive nature of selves in interaction, and from Williams and Stickley’s relational model of health [17], which situates well-being within social connection and creative engagement, we propose the analytic concept of “relational commons.” This concept refers to shared, situated spaces in which practices of conviviality, care, and well-being are collectively generated through ongoing interaction rather than delivered through formal intervention. Unlike social capital, which is often conceptualized as a resource that can be accumulated or mobilized, relational commons foregrounds process over possession and presence over outcome. It captures how health-related meanings emerge through everyday co-presence, invitation, and mutual attunement in ordinary settings, without requiring predefined roles, obligations, or therapeutic goals. The concept was developed inductively through engagement with participants’ descriptions of openness, playfulness, and shared presence, and is offered as an interpretive framework that links these situated experiences to broader relational theories of health and sociality.

Building on Antonovsky’s salutogenic model [13], this study foregrounds relational commons as a key analytical lens, emphasizing how conviviality and co-presence generate health. The Japanese notion of kakawari-shiro deepens this frame, situating it within a cultural context of invitation and aesthetic play. Related ideas in Creative Health [18, 19] and architectural theories of *ma* further illuminate the ethical and spatial dimensions of such relational practices.

### CBPR as relational and aesthetic practice

Several aspects of this project extend the conceptual boundaries of CBPR. First, it situates participation within an aesthetic and affective register. Many interactions occurred through art workshops and the Mobile Yatai—forms of “serious play” that enabled participants to express vulnerability and joy without the pressure of goal attainment. This resonates with what Gergen describes as relational being: [16] the idea that meaning and health arise from the generative interplay between selves rather than from individual attributes. Similarly, the “ethics of non-obligation” observed among participants echoes Japanese relational philosophies, where mutuality arises not from duty (*giri*) but from *omoiyari*—empathetic responsiveness to the presence of others [20, 21]. 

Such a perspective challenges conventional biomedical framings of health that privilege autonomy, control, and measurement. Instead, it aligns with contemporary discussions in the health humanities and creative health movements, which highlight imagination, narrative, and cultural participation as vital dimensions of health generation [22, 23]. By integrating these ideas, the YaNeSen project illustrates how CBPR can be enacted as a practice of care through culture—an approach where art and dialogue become health-promoting acts.

### The mobile yatai as a mediating device

The Mobile Yatai acted as a tangible mediator between the aesthetic and the relational dimensions of CBPR. As a movable, handcrafted structure combining hospitality and craftsmanship, it translated abstract ideas of co-presence into material form. The act of building and moving the yatai together turned streets into sites of collective authorship, transforming mobility itself into a practice of care [24, 25]. The stall’s design and movement fostered affective immediacy, lowering the social distance between residents and professionals while keeping participation voluntary and playful. In this sense, the yatai embodied a “convivial infrastructure” through which art, space, and health converged [19, 26]. 

### Social capital as living practice

The project’s evolution illustrates that *social capital* is not a static resource but a living, dynamic practice. Early ethnographic observations showed how temples, public baths, and narrow alleys functioned as “third places” [27] fostering everyday sociability. Over time, CBPR activities infused these spaces with renewed meaning, transforming them into “archives of relationship.” In Putnam’s terms [28], bonding and bridging forms of social capital were both strengthened: bonding among long-term residents through shared history, and bridging with newcomers through art and collaborative projects.

However, rather than instrumentalizing these ties for specific health outcomes, the project emphasized being together in ambiguity—a state of openness that allowed difference to coexist. This condition of ambiguous belonging may be particularly relevant in Japan’s aging urban centers, where social fragmentation and loneliness coexist with dense physical proximity [29]. The YaNeSen project suggests that trust in such contexts arises not from clarity of purpose but from shared tolerance for uncertainty.

### Reframing health: from individual to relational well-being

Participants’ narratives indicate a significant shift in how health was understood and experienced. Through participation in creative, non-hierarchical activities, individuals began to perceive health as relational, expressive, and existential. This aligns with the relational model of health proposed by Williams and Stickley [17], which posits that well-being is generated through meaningful social connection and creative engagement rather than the absence of illness. The arts-based components of the YaNeSen CBPR acted as micro-publics of healing where emotion, memory, and identity could be negotiated collectively [19]. 

Such findings also resonate with Antonovsky’s salutogenic framework [13], which shifts the focus from disease prevention to the creation of health through sense of coherence and meaningfulness. The YaNeSen participants’ reflections suggest that the project cultivated precisely these qualities: comprehensibility (understanding one’s place in the community), manageability (shared agency in collective projects), and meaningfulness (feeling that one’s presence matters).

### CBPR as “kakawari-shiro”: the space of invitation

A particularly distinctive contribution of this study is the conceptualization of CBPR as kakawari-shiro—literally, a “field of relationships.” Unlike typical participatory frameworks structured around explicit roles or deliverables, kakawari-shiro emphasizes presence, invitation, and porous boundaries. It is a space where participation is fluid, entry is casual, and engagement is sustained through affection and curiosity. In Japanese social philosophy, such openness echoes the concept of ma—the interval or empty space that enables connection and resonance [30, 31]. 

This framing situates CBPR not merely as a research methodology but as a cultural practice of co-being, where health emerges from shared rhythms of everyday life. It also provides an alternative to the neoliberal appropriation of community participation, where engagement is often instrumentalized for efficiency or cost reduction [32]. In contrast, kakawari-shiro values slowness, ambiguity, and care as ethical foundations of sustainable health promotion.

This relational and processual understanding of health also invites reflection on its political and economic implications. In particular, it sits in tension with neoliberal framings of health that emphasize efficiency, scalability, and cost-effectiveness. Within such frameworks, community-based initiatives risk being valued primarily for their capacity to deliver outcomes at lower cost or to compensate for gaps in formal health systems. In contrast, the *YaNeSen* project foregrounds practices of co-existence that are deliberately small-scale, open-ended, and relational, and therefore resistant to straightforward quantification. Acknowledging this tension is crucial to avoid the co-optation of Creative Health practices as forms of outsourced or informalized care, and to situate them instead as complementary—but not substitutive—within broader public responsibilities for health equity [33–35]. 

### Implications for urban health and policy

Urban Japan faces a paradox: while infrastructure and healthcare systems are highly developed, communities suffer from fragmentation and isolation. Traditional public health models often overlook the affective dimensions of place and belonging. The YaNeSen CBPR suggests that policies aiming at “community-based integrated care” (chiiki hokatsu care) may benefit from integrating cultural and relational strategies—including participatory art, storytelling, and shared public spaces—as integral components of health systems.

The Mobile Yatai further suggests that micro-scale and movable infrastructures can complement formal community-based integrated care systems, particularly by reaching populations who remain peripheral to institutional health promotion. While such initiatives may appear low-cost and flexible, their value lies not in efficiency or scalability per se, but in their capacity to cultivate relational presence, invitation, and trust over time. Importantly, these practices should not be understood as substitutes for public responsibility or institutional care, but as complementary relational infrastructures that operate alongside—and depend upon—broader commitments to health equity and social support.

Comparable arts- and community-based approaches to health have been reported in other contexts. In Japan, community arts festivals and neighborhood-based cultural initiatives have been shown to foster social connection and well-being, particularly among older adults and socially isolated populations [36, 37]. Internationally, Creative Health programs in the United Kingdom and other countries have emphasized cultural participation, social prescribing, and informal community spaces as responses to complex social determinants of health [22, 38, 39]. Situating the YaNeSen project alongside these initiatives highlights both shared principles—such as relational engagement and cultural participation—and its distinctive emphasis on everyday urban spaces, relational invitation, and the absence of predefined therapeutic goals.

In this sense, the YaNeSen project contributes to the field of Creative Health by offering a culturally grounded and relationally oriented model of participatory health creation. By demonstrating how low-cost, art-based infrastructures can complement—rather than substitute for—formal public health systems, the project reframes culture not as an instrument for behavior change but as a relational and aesthetic medium through which health can emerge. Such micro-scale convivial infrastructures, sustained by everyday encounters and rooted in local culture, illustrate how Creative Health initiatives can emerge from everyday cultural practices, while remaining connected to broader public and institutional responsibilities, informing urban health policies that seek to alleviate loneliness and social fragmentation through cultural participation.

In sum, the YaNeSen CBPR demonstrates how ethnographically grounded, art-based participation can cultivate relational well-being in urban Japan. By linking everyday encounters to broader frameworks of salutogenesis and Creative Health, the study offers a model of kakawari-shiro—a culturally embedded form of participatory health creation where openness, ambiguity, and aesthetic engagement are not limitations but sources of vitality.

### Limitations and future directions

This study is intentionally framed as a qualitative, single-site CBPR case study conducted in a culturally specific urban district. As such, the findings are not intended to be statistically generalizable, but rather to offer a contextually grounded interpretation of relational health practices in an urban Japanese setting. The sustained engagement over time provides detailed insight into the processual and relational dynamics through which community health can be cultivated.

It should also be noted that no participants explicitly reported strongly negative experiences during the follow-up interviews. Individuals who disengaged earlier or held more critical perspectives may therefore be underrepresented, potentially contributing to a more affirmative account of participation. This limitation reflects a common challenge in longitudinal, community-based qualitative research and should be considered when interpreting the findings.

Future research may explore how similar forms of relational invitation and convivial infrastructure are enacted in different cultural and institutional settings. Methodologically, integrating arts-based evaluation [40] and participatory visual methods could further illuminate how affect and imagination shape community well-being. Quantitative indicators such as social participation, loneliness, or life satisfaction may complement these qualitative insights, enabling mixed-methods evaluation of relational health.

## Conclusion

The YaNeSen CBPR exemplifies how health can be co-created through culture, dialogue, and shared experience. By foregrounding presence over productivity and invitation over obligation, the project nurtured a living ecology of relationships that redefined what it means to be well together. The Mobile Yatai served as both a symbolic and material vehicle for this process—transforming ordinary streets into convivial spaces where care could be enacted through everyday gestures of hospitality and curiosity. Its mobility, informality, and aesthetic appeal enabled encounters that transcended professional boundaries and reconnected health with the rhythms of local life.

More broadly, the YaNeSen project extends the conceptual boundaries of CBPR by demonstrating that participation can be cultural as well as procedural, aesthetic as well as ethical. It highlights the potential of small-scale, art-based, and relational infrastructures to complement formal health systems and address urban isolation through creative engagement. In an era of demographic aging and social fragmentation, such relational commons may offer a blueprint for a more inclusive and humane public health—one rooted not in intervention, but in convivial coexistence and mutual flourishing.

## Data Availability

The qualitative data generated and analyzed during this study are not publicly available to protect participant confidentiality but are available from the corresponding author upon reasonable request.
